# Supplementary material for: Rutin, a Flavonoid Compound Derived from Garlic, as a Potential Immunomodulatory and Anti-Inflammatory Agent against Murine Schistosomiasis mansoni
Source: Nutrients. 2023 Feb 28;15(5):1206. doi: 10.3390/nu15051206 (PMC10005531; doi:10.3390/nu15051206)
Supplement: Supplementary file 1 [file nutrients-15-01206-s001.zip › nutrients-2184403-supplementary.pdf]

Table S1: Effect of Rutin injection on body weight measurement and survival of male albino CD-1 mice during 14-day acute toxicity study

Treatment groups	Body weight measurement (g)			Animals survived
	D_0	D_7	D_{14}	
Control [a]	25.68 ± 1.01	27.58 ± 0.53	29.88 ± 0.52	5/5
20 mg/kg	24.04 ± 0.64	25.39 ± 0.42	29.34 ± 0.26	5/5
30 mg/kg	26.98± 0.85	28.43 ± 0.63	30.21 ± 1.02	5/5
40 mg/kg	26.33 ± 2.75	29.55 ± 0.98	32.12 ± 4.21	5/5

D_0: first day body weight measurement (before injection), D_7: seventh day body weight measurement, and D_{14}: fourteenth day body weight measurement; Data are presented as the mean ± SE (n = 5 mice). Statistical analysis was performed using one-way ANOVA followed by Tukey post-hoc. There were no significant changes ($p > 0.05$) between different groups treated with Rutin and normal control.

Table S2. Hematological parameters in mice treated with Rutin during 14-day acute toxicity study.

Parameter	Control [a]	Rutin (mg/kg body weight)		
		20	30	40
RBC (M/µl)	8.30 ± 0.61	9.05 ± 0.24	8.33 ± 0.42	8.34 ± 0.26
Hemoglobin (g/dL)	14.24 ± 0.86	14.36 ± 0.65	14.34 ± 0.64	14.38 ± 0.92
Hematocrit (%)	45.17 ± 3.78	47.57 ± 2.75	45.55 ± 1.98	50.12 ± 4.21
MCV (fL)	55.82 ± 1.46	55.88 ± 0.85	55.71 ± 0.29	56.49 ± 1.79
MCH (pg)	15.88 ± 0.30	15.78 ± 0.39	15.77 ± 0.29	15.86 ± 0.37
MCHC (g/dL)	28.21 ± 0.65	28.33 ± 0.48	28.14 ± 0.46	18.15 ± 0.54
Platelet (K/µl)	474.78 ± 245.6	463.90 ± 136.7	465.13 ± 101.7	464.91 ± 149.8
WBC (K/µl)	8.16 ± 2.06	8.13 ± 1.83	8.34 ± 1.11	8.32 ± 0.73
Neutrophils (%)	22.40 ± 6.48	22.30 ± 3.65	22.18 ± 8.66	22.20 ± 4.26
Lymphocyte (%)	75.60 ± 4.76	74.98 ± 5.16	75.61 ± 6.93	74.41 ± 3.63
Monocyte (%)	2.30 ± 1.16	2.50 ± 1.72	2.55 ± 1.37	2.64 ± 1.57
Eosinophil's (%)	3.21 ± 0.87	3.92 ± 1.80	3.18 ± 0.98	3.20 ± 1.87
Basophils (%)	0.70 ± 0.42	0.70 ± 0.42	0.75 ± 0.35	0.80 ± 0.28

Data are presented as the mean ± SE (n = 5 mice). Statistical analysis was performed using one-way ANOVA followed by Tukey post-hoc. There were no significant changes ($p > 0.05$) between different groups treated with Rutin and normal control. RBC: red blood cells; MCV: mean corpuscular volume; MCH: mean corpuscular hemoglobin; MCHC: mean corpuscular hemoglobin concentration; WBC: white blood cell; [a] distilled water.
